# Evolution of strategic cooperation

**DOI:** 10.1002/evl3.164

**Published:** 2020-02-25

**Authors:** P. G. Madgwick, J. B. Wolf

**Affiliations:** ^1^ Milner Centre for Evolution, Department of Biology and Biochemistry University of Bath Bath BA2 7AY United Kingdom

**Keywords:** Cooperation, group selection, kin selection, public goods, social behavior

## Abstract

Group‐beneficial behaviors have presented a long‐standing challenge for evolutionary theory because, although their benefits are available to all group members, their costs are borne by individuals. Consequently, an individual could benefit from “cheating” their group mates by not paying the costs while still reaping the benefits. There have been many proposed evolutionary mechanisms that could favor cooperation (and disfavor cheating) in particular circumstances. However, if cooperation is still favored in some circumstances, then we might expect evolution to favor strategic cooperation, where the level of contribution toward group‐beneficial behavior is varied in response to the social context. To uncover how and why individuals should contribute toward group‐beneficial behavior across social contexts, we model strategic cooperation as an evolutionary game where players can quantitatively adjust the amount they contribute toward group‐beneficial behavior. We find that the evolutionarily stable strategy (ESS) predicts, unsurprisingly, that players should contribute in relation to their relatedness to the group. However, we surprisingly find that players often contribute to cooperation in such a way that their fitness is inverse to their relatedness to the group such that those that contribute to cooperation end up with the same return from group‐beneficial behavior, essentially removing any potential advantage of higher relatedness. These results bring to light a paradox of group‐beneficial cooperation: groups do best when they contain highly related individuals, but those with the highest relatedness to the group will often have the lowest fitness within the group.

**Impact Summary**

What motivates organisms to contribute toward group‐beneficial behaviors? This question is challenging for evolutionary theory because such cooperative behaviors typically incur a personal cost, whereas their benefits are open to exploitation by everyone in the group—including noncontributors. To address this fundamental problem, we develop a simple game theoretical model of cooperation through “public goods.” We allow the players in the game to strategically adjust their contributions to cooperation to understand how and why individuals should make costly contributions to the potential benefit of others. Our model elucidates how relatedness within groups molds cooperative strategies, revealing a paradoxical outcome that those with the highest relatedness to a group end up with the lowest fitness in the group. We also identify a surprising result, where individuals who contribute to cooperation do so in a way that leaves them with an equal fraction of the group's fitness, even if they have different levels of relatedness to the group. In addition to providing a clear framework that can be used to make testable empirical predictions, our model also provides a possible mechanism that can explain the maintenance of genetic variation in cooperative systems (which is captured in the long‐standing problem of “Crozier's paradox”).

## Introduction

Group‐beneficial social behavior is near‐universal across the diversity of life, ranging from communal care in vertebrates (Clutton‐Brock 2009) through self‐sacrificial defensive behaviors in eusocial insects (Wilson and Hölldobler 1990) to secreted metabolic molecules in microorganisms (West et al. 2006). Despite covering wildly different taxa and ecologies, these types of social behaviors all share a common puzzle: the benefits are available to everyone in the group, and yet their costs are borne by the particular individuals that perform them (Wilson 1977; Frank 1995; Rankin et al. 2007; Johnstone and Rodrigues 2016). Why should an individual pay the cost of group‐beneficial behavior rather than “cheating” their group mates by not paying their fair share of the costs? In general, the evolutionary problem of cheating is expected to lead natural selection to disfavor group‐beneficial behaviors (Frank 2003; Foster 2004; Travisano and Velicer 2004; West et al. 2007; Bourke 2011), but there are numerous mechanisms that tip the balance of selection in their favor within specific contexts, such as high relatedness between social partners (Hamilton 1964), strong reciprocal interactions (Trivers 1971), and enforcement mechanisms like punishment (Clutton‐Brock and Parker 1995) or policing (Frank 1995).

Logically, if cheating is favored in some circumstances and not in others, then we might expect selection to favor “strategic” cooperation, where individuals modulate the amount they contribute toward group‐beneficial behavior in different situations. Although learning could potentially play a role in strategic cooperation, here we are focused on genetic strategies that evolve to optimally adjust the level of cooperation by an individual to suit its social context through phenotypic plasticity. Such strategic cooperation could appear as facultative cooperation, where individuals contribute toward group‐beneficial behavior when it is profitable (in terms of fitness) and withhold their contributions when it is not. However, strategic cooperation need not appear as an all or none pattern of behavior. Indeed, an individual would often do better by strategically adjusting their contribution to cooperation in response to the relative costs and benefits. In this way, strategic cooperation is not a mechanism that prevents or avoids cheating but, rather, an adaptation that allows individuals to maximize their fitness within any given social context (whether this means making contributions toward cooperation or free‐riding on the contributions of others).

Strategic cooperation is most likely to evolve when social contexts are highly variable, and hence natural selection can favor different levels of cooperation in different situations. For example, pairs of female house mice have been shown to adjust their contribution to communal care in response to unequal litter sizes, increasing the time spent caring for the group's offspring when their own offspring make up a larger fraction of the group (Ferrari et al. 2016). Similarly, soldier‐producing aphids increase their contributions toward gall defense behavior when in highly related groups, which comes at a cost to their personal reproduction (Abbot et al. 2001). Likewise, strains of a social amoebae modulate the fraction of cells that differentiate into non‐reproductive stalk cells (that help the remaining spores cells to disperse) dependent upon their frequency within the group (Madgwick et al. 2018). These examples, from cooperatively breeding vertebrates, eusocial insects, and social microorganisms, indicate that strategic cooperation can occur across diverse taxa and that it need not require complex cognitive skills. Such strategic cooperation could vary in response to some signal/cue that indicates relatedness through a greenbeard or kin recognition gene (Gardner and West 2010; Madgwick et al. 2019; see Model and Results). Consequently, strategic cooperation may help explain why group‐beneficial behaviors persist throughout nature, despite the potential advantages of cheating. Strategic cooperation may also explain the “missing cheaters” phenomenon—why obligate cheater types are so surprisingly rare in nature (Gilbert et al. 2007). Instead, if individuals are able to show flexible social strategies where they strategically modulate their level of cooperation to best exploit their social environment, we would expect any individual to appear to be a contributor or a free‐rider (i.e., appear to be a cheater) in different situations.

To uncover how individuals should change their contributions toward group‐beneficial behavior in different social contexts, we develop a general theoretical framework to model strategic cooperation as a flexible social strategy where individuals can quantitatively adjust their contributions toward group‐beneficial “public goods.” We identify the evolutionarily stable strategy (ESS), which represents a quantitative pattern of how much an individual should contribute toward cooperation across social contexts to maximize their fitness. To elucidate the factors that shape the ESS, we examine the evolutionary logic of strategic cooperation within different conceptual frameworks (kin and group selection).

## Model and Results

We model cooperation through a group‐beneficial behavior that generates a “public good” that benefits all group members (Hamburger 1973; Fox and Guyer 1978; Dionisio and Gordo 2006; Hauert et al. 2006; Frank 2010; Archetti and Scheuring 2011; Gavrilets 2015). Hence, our analysis does not include cooperative behaviors that are limited to one‐way transactions, such as where an actor either helps or harms a recipient. Across different scenarios, the prevailing feature of public goods is that they are costly to produce, leading to a trade‐off between the production of public goods and other fitness‐related traits (Haldane 1932; Wright 1945; Maynard et al. 1964; Wilson 1975; Frank 1995). Cooperation through the production of costly public goods is widespread, but the form of cooperation can vary widely. Public goods could refer to a physical product, such as secreted molecules in microbes that come at an energetic cost to the cells that produce them (which presumably reduces the growth rate of those cells), while being available to all cells in the local environment (West et al. 2006). For example, iron‐scavenging siderophores in the bacterium *Pseudomonas aeruginosa* (Griffin et al. 2004) and extracellular enzymes within biofilms in the virus *Vibrio cholerae* (Drescher et al. 2014) are energetically costly to produce and increase the fitness of all cells in the local area. Similarly, individuals can cooperate in the production of physical structures that act as public goods, such as the fruiting body stalk that facilitates dispersal in the social amoeba *Dictyostelium discoideum* (Madgwick et al. 2018) and social bacteria *Myxococcus xanthus* (Velicer et al. 2000), which comes at a clear cost to the cells that are sacrificed for its production. Public goods could also arise from behavioral services (Foster 2004), such as vigilance in meerkats (Santema and Clutton‐Brock 2013) and monkeys (Gaynor and Cords 2012), which is costly because it competes with time that an animal could use for other purposes and may also expose the individual to direct danger, while benefiting all individuals in the local area who can respond to an alarm call (signaling an approaching predator). Likewise, cooperatively breeding animals may often generate public goods through indiscriminate communal care (Riehl 2013; Ferrari et al. 2015) that benefit all the young in a communal nest, while coming at a cost to an individual's own fecundity. Thus, given that there is a broad range of scenarios where individuals cooperate through public goods in nature, we do not construct our model to match any specific biological scenario. Instead, we implement a generalized framework (building on Madgwick et al. 2018) that considers the problem from the perspective of competing genetic variants that are maximizing their transmission, which can be adapted to fit a large range of different systems.

We build a game‐theoretic model where the “players” represent different genetic variants (i.e., coreplicons; Cosmides 1981). Importantly, players are not equivalent to individuals but, rather, can be represented by multiple individuals who share copies of the same single genetic variant. Consequently, we do not explicitly focus on what individuals should do to maximize their fitness, but instead focus on what genes should do to maximize their fitness. This perspective is equivalent to considering how individuals should behave to maximize their inclusive fitness, but allows for simpler and more direct accounting of fitness given that we assume that the evolutionary players are the competing genetic variants. By adopting this approach, we can still examine the consequences of the evolution of strategic cooperation for individual behavior because individuals should behave according to the interests of the genetic variant controlling the behavior (and hence individuals will act to maximize the inclusive fitness of that variant).

From this perspective, we consider strategic cooperation in response to a genetic variant's frequency, which requires genetic variants to be able to assess their presence or absence in a group and change their level of cooperation in response to their overall frequency in the group (which is equivalent to assessing their relatedness to the group). This scenario could correspond to the presence of a greenbeard gene that responds to group members sharing the same greenbeard genetic variant (Grafen 1985), which could arise when the greenbeard gene encodes a signal‐receptor protein that regulates the level of cooperation (Haig 1996; Springer et al. 2011; Madgwick et al. 2019), of which there are numerous examples across taxa (Gardner and West 2010; Madgwick et al. 2019). Alternatively, this scenario could correspond to the presence of a kin recognition gene that responds to the relatedness across the genome (Grafen 1985), which also has numerous examples across taxa (Penn and Frommen 2010). The difference between the evolutionary consequences of a greenbeard and kin recognition gene is how they affect the relatedness profile across the genome (Grafen 1985; Gardner and West 2010; Madgwick et al. 2019) but, as we are only considering strategies associated with genetic variants at a single locus, these two possible sources of relatedness are indistinguishable in our framework. In principle, strategic cooperation could also rely upon behavioral adjustment through reciprocity that relies upon expressing a shared phenotype, but we do not consider this case because outcomes can be dependent upon different possible forms of partner feedbacks (Queller 2011). Therefore, we assume a genetic recognition mechanism, such that genetic variants are either greenbeards or kin recognition genes, to restrict our attention to how natural selection shapes the genetic evolution of strategic cooperation.

We consider groups of *N* players, where each (*i* ^th^) player is present at a particular frequency $p_{i}$ within the group (such that $\sum_{i\;=\;1}^{N}p_{i}=\;1$). We assume that a player's frequency within a group is independent of their strategy and that variation in the frequency of a player across groups is caused by some process that allows players to experience a range of group compositions (and so we examine the consequences of variation in group composition, not the underlying causes of the variation). In the absence of any contribution to public goods, all players within a group have an equal baseline fitness value of 1, which can be viewed as the budget from which they can make a contribution toward public goods. In this way, we assume the cost of producing public goods is measured in units of fitness and represents a separate component of fitness, and hence making a contribution comes at the expense of a player's potential fitness through other traits. For example, a player's resource budget might determine the potential number of eggs they could produce, and a contribution toward public goods would incur a cost to the production of eggs. Players contribute a proportion ($x_{i}$) of this potential fitness into public goods ($0\leq x_{i}\leq 1$), which results in a reduction in fitness of *c* per unit contributed, making the total cost $cx_{i}$. After contributing to public goods, a player has a residual fitness of $1-cx_{i}$ before accounting for any fitness benefits from the public goods. Because contributions are measured in units of fitness, logically *c* would equal 1, but we retain a *c* parameter for generality (in capturing scenarios where fitness declines faster or slower per unit investment, which could capture strategic constraints) and symmetry with *b* (to enable comparisons relating to the relative cost‐to‐benefit ratio). The benefit from public goods depends on the total collective contribution made by all members of a group ($x_{G}=\sum_{i=1}^{N}p_{i}x_{i}$) because the public goods benefit everyone, regardless of which player “paid” to produce them. Each unit of collective contribution gives a benefit of *b* in units of fitness and represents another component of fitness, which makes the total impact of collective contribution to public goods on a player's fitness $1+bx_{G}$. Although it is possible that some systems might show a nonlinear relationship, such as those with diminishing or accelerating benefits from public goods, the general patterns of results would hold under these other relationships insofar as they are monotonically increasing functions (and, moreover, even nonlinear relationships may be quasi‐linear in the evolutionarily relevant range of contributions).

We assume that a player's realized fitness (after accounting for the costs and benefits of public goods) is determined by the product of the costs from contributing to $\left(1-cx_{i}\right)$ and the benefits from the availability of public goods $\left(1+bx_{G}\right)$:

(1) $$\omega_{i}=\;\left(1-cx_{i}\right)\left(1+bx_{G}\right).$$

See Table 1 for a summary of model notation. As in similar models (Frank 1995; Frank 1996; Hauert et al. 2006; Frank 2010; Gavrilets 2015), fitness is multiplicative (rather than additive) because the costs and benefits represent different fitness components. This is logical because it implies that fitness reflects the realized benefit of public goods, which depends on both the fitness cost paid for their contribution to and benefit arising from public goods. This reflects the fact that it is through the residual potential fitness after contributing that players gain the fitness benefit from the public goods, and so contributing to public goods reduces a player's potential benefit from the public goods. Consider again the example where costs of contributing to public goods reduce the number of eggs a player produces. If players sacrifice egg production because they use their energetic resources for public goods that increase survival, such as through antipredator vigilance (where alarm calls benefit all members of their group), then it is logical that total fitness is the product of the residual fecundity (which is reduced owing to the energy sacrificed for antipredator vigilance) and survival (which is higher because vigilance reduces predation). Importantly, in contrast to the commonly used framework that assume additivity of linear costs and benefits, the multiplicative model of fitness we use (eq. 1) captures the nature of biological trade‐offs, where players must make strategic decisions over how to allocate limited resources to different components of fitness. As a consequence of such trade‐offs, selection will typically favor some intermediate level of investment into public goods that maximizes a player's overall fitness (see below). In contrast, a model in which linear costs and benefits are additive (e.g., Hauert et al. 2006; Archetti and Scheuring 2011; Gavrilets 2015) would not capture the biology of resource allocation trade‐offs and, as a result, would lead to a scenario where we could only see a pattern of facultatively “all‐or‐none” cooperation (wherein, if benefits outweigh costs, players should invest, and, if they do not, players should not). Consequently, in an additive model with linear costs and benefits, players would not quantitatively adjust their strategy in response to what other players do, whereas in the multiplicative model that we consider with trade‐offs between fitness components, the optimal level of cooperation is expected to depend on the level of cooperation by others (i.e., the social context). Despite the apparent conceptual difference between these additive and multiplicative formulations of the fitness associated with investment in public goods, however, the two approaches are expected to be logically consistent because they essentially represent different scenarios that can be used to address different questions. The additive framework would be appropriate if we wanted to evaluate whether a player should invest an amount $x_{i}$ into public goods, where we would be asking whether the benefit of such an investment outweighs the cost given the social context (see “The kin selection perspective”). However, if wanted to evaluate how much a player should invest (i.e., what value of $x_{i}$ maximizes the player's fitness), we would need to use a model that captures the trade‐off associated with allocation of limited resources, such as the multiplicative model. Because we wish to answer the latter question—how much should players contribute to public goods—we therefore use the latter approach.

**Table 1:** Notation for the model

Parameter	Definition
*b*	Benefit (per unit of collective investment) to the group from public goods
*c*	Cost (per unit of investment) to a player from public goods
*N*	Number of players in the group
$\omega_{i}$	Fitness of the *i* ^th^ player
$p_{i}$	Frequency of the *i* ^th^ player
$x_{i}$	Investment strategy of the *i* ^th^ player
$x_{G}$	Collective investment of all players in the group
${\hat{x}}_{i}$	Evolutionarily stable strategy (ESS) with respect to the *i* ^th^ player
*n*	Number of players in the group that contribute to public goods
$\bar{p}$	Average frequency of all contributing players in the group
${\hat{x}}_{G}$	Coevolutionarily stable strategy (coESS) of collective investment by a group of players each pursuing the ESS

Although a player's fitness depends on the total collective contributions to public goods made by the group, to understand why a player personally contributes we need to consider their marginal impact on their own fitness owing to their own contribution to collective investment: $1+bp_{i}x_{i}$. Given that contributing to public goods comes at a cost with respect to potential fitness $1-cx_{i}$, any contribution by a player must ultimately increase their fitness above their baseline fitness, which can be shown by considering $\omega_{i}>1$, which (using eq. 1) rearranges to $\left(1+bp_{i}x_{i}\right)>1/\left(1-cx_{i}\right)$. This perspective emphasizes the dilemma of cooperation through public goods: the cost is paid directly, but benefits are diluted across the group, and therefore a genetic variant only sees a return on its contribution to public goods in relation to its frequency in the group.

The biologically relevant solution for players’ investment strategies is the ESS, which describes the best strategy that a player can adopt in an equilibrium population of strategies that have evolved by natural selection (Maynard Smith 1974). An additional level of complexity for the ESS of a continuously variable social behavior is that the ESS must describe a player's strategy across the full range of social contexts—and, critically, with respect to whatever information causes that player to modulate their strategy. Here, we focus our attention on the most unconstrained case, where the ESS describes what proportion of a player's fitness budget they sacrifice to produce public goods across groups with any number of players and any distribution of frequencies of players within the group. Thus, we assume that players have access to perfect information about their social context. By considering players to be genetic variants, this assumption implies that they are able to perfectly measure their frequency within the group (e.g., using a signal) and can then modify their contribution toward public goods in response (which is a necessary requirement of any frequency‐dependent strategy). Although the assumption of perfect information allows for the derivation of simple and tractable solutions to the model, the qualitative results hold in the case where individuals make some degree of error in their assessment of their social context (with the amount and pattern of error dictating the extent to which we expect a biological system to match the results of the perfect information scenario). We further assume that each player's response to a given social context is independent of its response to other social contexts, which simply means that the pattern of cooperation across social contexts is genetically unconstrained and can evolve to maximize a genetic variant's fitness (Maynard Smith 1976; Grafen 1984; Parker and Maynard Smith 1990; Kirkpatrick and Gomulkiewicz 1992).

Using optimality assumptions (Maynard Smith 1976; Grafen 1984; Parker and Maynard Smith 1990; Kirkpatrick and Gomulkiewicz 1992), we can solve the ESS (which we denote ${\hat{x}}_{i}$) by finding the pattern of contribution to public goods that maximizes a player's fitness ($\omega_{i}$) given their frequency in a group. We refer to this pattern as their “investment strategy” to reflect the fact that the level of contribution to public goods is that which maximizes the benefit against the costs. Given that a player's investment strategy is defined as the proportion of their potential fitness that they devote toward public goods, given an equilibrium quantity of collective investment by the group (${\hat{x}}_{G}$) as, the ESS can be solved using equation (1) by setting its derivative ($d\omega_{i}/dx_{i}$) equal to zero and rearranging (see also Supporting Information 1):

(2) $${\hat{x}}_{i}=\frac{p_{i}b-c-bc{\hat{x}}_{G}}{p_{i}bc}.$$

In broad terms, the ESS shows that a player contributes a larger proportion of their fitness budget toward public goods when they are at a higher frequency in a group ($p_{i}$), but their exact level of investment can depend on the behavior of others (which is reflected in the dependence of individual investment on group investment). Because the solution to equation (2) depends on the level of investment by the group (${\hat{x}}_{G}$; i.e., including the focal player), it does not represent a closed solution (because the investment made by each group member relies on the level made by all other group members, and hence the values are all interdependent). Therefore, to solve a player's ESS level of investment toward public goods, the expression in equation (2) must be resolved simultaneously for all players in a group.

Because equation (2) can be negative, it is only possible to solve an analytical expression for the ESS by resolving whether each player contributes or not (see Supporting Information 1). For two players, the ESS is resolved by four conditional expressions (from the combinations of ${\hat{x}}_{1}\geq 0$ or ${\hat{x}}_{1}<0$ and ${\hat{x}}_{2}\geq 0$ or ${\hat{x}}_{2}<0$), but the number of conditional expressions needed to describe the ESS increases exponentially with additional players. Consequently, this analytical description of the ESS is not very informative. Therefore, instead, it is possible to derive a more informative analytical description of the ESS for any number of players using a novel approach that we refer to as the coevolutionarily stable strategy (coESS) (but see Eshel [1985] for a similar usage). The coESS describes the quantity of collective investment by the group (${\hat{x}}_{G}$) when each player contributes toward public goods at the level of the ESS for their frequency. The coESS represents the group‐level ESS, which we use because it enables the collective properties of multiple players that invest at the ESS to be uncovered—and in turn, this greatly clarifies the variables underpinning an individual player's ESS. Thus, the coESS is the sum of the contributions made by all group members toward public goods at equilibrium across the full range of possible frequencies of players within groups (${\hat{x}}_{G}=\sum_{i=1}^{n}p_{i}{\hat{x}}_{i}$). Within any particular group, not all players necessarily contribute toward public goods at the level given by the coESS because, under many conditions, one or more players pursuing the ESS do not contribute at all given their frequency. Consequently, at the coESS the total investment depends on a subset of investing players; for any particular group, we can therefore define that there is a set of *n* players who invest, which we refer to as the “contributors,” and N−n players that do not, which we refer to as the “free‐riders.” We can express the solution in terms of the mean of the frequencies of all contributors ($\bar{p}=\frac{1}{n}\;\sum_{i\;=\;1}^{n}p_{i}$), which also gives the proportion of the group that contributes: $\bar{p}n$ (making the proportion of free‐riders in a group $1-\bar{p}n$). The total investment for a group is, therefore, the sum of the investment by contributors (${\hat{x}}_{G}=\sum_{i=1}^{n}p_{i}{\hat{x}}_{i}$), which we can describe with greater specificity using these new terms (see also Supporting Information 1):

(3) $${\hat{x}}_{G}=\frac{n\;\left(\bar{p}b-c\right)}{\left(n+1\right)bc}.$$

In broad terms, the coESS shows that a group of players invest more into public goods when there are fewer contributors at a higher average frequency. Because the coESS reflects the ESS behavior for a group of contributors, we can substitute the coESS into the ESS (eq. 2) to arrive at a more exact analytical solution for the ESS level of investment by a contributor:

(4) $${\hat{x}}_{i}=\frac{p_{i}b-c-n\;\left(\bar{p}-p_{i}\right)b}{p_{i}\;\left(n+1\right)bc}.$$

This solution to the ESS is only fully resolved for a particular group once players are classified as being one of the *n* contributors or N−n free‐riders. A player can be categorized as a contributor or free‐rider based on their frequency in a group (see Supporting Information 1; Table 2).

**Table 2:** The categorization of players as free‐riders or contributors. All players within a group can be classed as a contributor or free‐rider depending on their frequency. The frequency threshold that separates the two classes depends on the costs (*c*) and benefits (*b*) of investment into public goods. Once classified, players can be assigned an investment level (${\hat{x}}_{i}$) and expected fitness (***ω_i_***)

Investment class	Frequency limit	Investment level	Fitness
Free‐rider	$p_{i}<\frac{\bar{p}nb+c}{b(n+1)}$	${\hat{x}}_{i}=\;0$	$\omega_{i}=\frac{\left(\bar{p}nb+c\right)}{c(n+1)}\;$
Contributor	$p_{i}\geq \frac{\bar{p}nb+c}{b(n+1)}$	${\hat{x}}_{i}=\frac{p_{i}b-c-n\left(\bar{p}-p_{i}\right)b}{p_{i}bc\left(n+1\right)}\;$	$\omega_{i}=\frac{{\left(\bar{p}nb+c\right)}^{2}}{p_{i}bc{\left(n+1\right)}^{2}}\;$

There is a fascinating and unexpected property of the ESS: each of the *n* contributors has equal transmission to the next generation, despite having different starting frequencies. Transmission refers to the number of copies of that particular genetic variant that are passed to the next generation, which is calculated as their frequency‐weighted fitness ($p_{i}\omega_{i}$). Thus, in real terms, equal transmission would mean that genetic variants produce the same proportion of the progeny from that group, rather than (as might otherwise be assumed) a player at higher frequency in a group producing a larger proportion of all progeny produced by the group. This result can be demonstrated by examining the frequency‐weighted fitness of any contributor via substituting the expression for the ESS (eq. 4) into the expression for a player's fitness (eq. 1), which simplifies to $p_{i}\;\omega_{i}=\;\left(c/b\right){\left(1+b{\hat{x}}_{G}\right)}^{2}$ (Table 2). This expression means that contributors must make unequal contributions to public goods that wipe out the differences in their starting frequencies, which necessarily means that those at higher frequency are investing more (and hence are paying a larger fitness cost) than those at lower frequency. Although all contributors end up with equal transmission to the next generation, their transmission is always higher than free‐riders (i.e., the individual contributors each account for a larger proportion of all progeny produced by members of the group than do each of the free‐riders). However, free‐riders have higher fitness than contributors because they do not pay for any of the costs of public goods and yet receive the same benefit as contributors.

## The Kin Selection Perspective

We can understand the logic of why and how much players contribute to public goods by relating the model results to the kin selection perspective. For this, we can examine the conditions where a player should contribute toward the production of public goods rather than being a free‐rider, which we can express by finding the conditions that satisfy the inequality for when the ESS level of investment is greater than zero:

(5) $$p_{i}b-c-n\;\left(\bar{p}-p_{i}\right)b>0.$$

The first two parts of this expression ($p_{i}b-c$) represent a simple form of Hamilton's rule (Hamilton 1964; Charnov 1977), whereas the third part $-n(\bar{p}-p_{i})b$ captures how a player should adjust their investment strategy in response to the investment by their group mates. The expression $p_{i}b-c$ captures the direct “profitability” of investment into public goods (in terms of fitness), where each unit of investment results in a reduction in fitness of *c* and a benefit in terms of $p_{i}b$. A player's frequency is multiplied by the benefit term because this term scales how much a player's contribution toward collective investment is able to impact that player's own fitness. For example, a player at low frequency in a group is largely incapable of affecting their own fitness through their contribution to public goods, regardless of how much they might invest (because their contribution is diluted through the group in relation to their frequency). This component of equation (5) ($p_{i}b-c$) directly matches the classic form of Hamilton's rule because the frequency of a player in the group ($p_{i}$) also represents its relatedness to the group (which includes itself). The last component of equation (5) ($-n(\bar{p}-p_{i})b$) captures the effect of other players’ investment on the motivation for the focal player to contribute toward public goods. When a player is at a higher frequency than the average frequency of contributors, this last term will be positive and have no impact on the conditions because the first condition ($p_{i}b-c>0$) has to be met for that player to be motivated to invest in the first place. However, if a player is at a frequency that is below the average frequency of contributors, this last term is negative and indicates that such players will have a greater incentive to free‐ride on the public goods produced by others (and hence reduce their own contribution). In this way, this term ($-n(\bar{p}-p_{i})b$) captures the conflict that arises between players, as they reduce their investment in response to the investment made by other players (cf. Boyd and Richerson 1988; Frank 1998). Therefore, overall, whether a player contributes toward the production of public goods or free‐rides depends on a combination of the potential profits of investing from a simple cost‐benefit analysis and also the impact of social conflict.

We can also examine the impact of these two factors—the simple cost‐benefit analysis that determines the profitability of investing in public goods and the impact of social conflict that can disincentivize contributing, in terms of the quantitative level of investment into public goods that is favored. The ESS (eq. 2) can be rearranged into two components that reflect these factors by splitting the collective investment by the group (the coESS) into contributions from the focal player and all other players (${\hat{x}}_{G}=p_{i}\;{\hat{x}}_{i}+p_{-i}{\hat{x}}_{-i}$), which are separated into terms by the square brackets (see Supporting Information 1):

(6) $${\hat{x}}_{i}=\left[\frac{1}{2}\left(\frac{1}{c}-\frac{1}{p_{i}b}\right)\right]\;-\left[\frac{1}{2}\left(\frac{p_{-i}{\hat{x}}_{-i}}{p_{i}}\right)\right].$$

The first term in square brackets contains the Hamilton's rule cost‐benefit balance that depends solely on a player's frequency or relatedness to the group ($p_{i}$). The second term in square brackets contains the effect of other players’ investment and, because this bracket is taken away from the first, other player's investment always acts as a “conflict load” in decreasing the quantity of investment that a player contributes compared to the simple Hamilton's rule. Therefore, although a player's quantitative contributions toward cooperation are not determined by a familiar Hamilton's rule, the quantity of investment is shaped by the same factors that determine whether a player is a contributor or free‐rider (see Supporting Information 2 for an examination of a marginal Hamilton's rule that can be used to solve the ESS, albeit encountering the same relationship between the number of conditional expressions needed to describe the ESS and the number of players, as described above).

## Group Selection Perspective

We can also understand the logic of why and how much players contribute to public goods by relating the model results to the group selection perspective. For this, we can partition a player's fitness into the product of within‐ ($u_{i}$) and between‐group ($u_{G}$) components ($\omega_{i}=u_{i}\;u_{G}$). This partitioning is in keeping with group selection models (Haldane 1932; Wright 1945; Maynard et al. 1964; Wilson 1975), even though we are not modeling selection on groups per se because we do not consider within‐ and between‐group competition (but rather the ESS that is selected to maximize a player's fitness within any given group context). Nevertheless, we can derive a within‐group component of fitness to describe what proportion of the benefits from public goods a player receives compared to their group mates, whereas the between‐group component of fitness describes the overall magnitude of the benefits of public goods to the group. This partitioning yields an expression for within‐group fitness:

(7) $$u_{i}=\frac{p_{i}\;\left(1-c{\hat{x}}_{i}\right)}{1-c{\hat{x}}_{G}}.$$

This indicates that a player has maximal within‐group fitness when they do not invest at all (as long as others invest, otherwise all players would have equal fitness). The partitioning also yields an expression for between‐group fitness, which is defined as the sum of the fitness of the *N* players that compose the group ($u_{G}=\sum_{i=1}^{N}\omega_{i}$):

(8) $$u_{G}=\;\left(1-c{\hat{x}}_{G}\right)\left(1+b{\hat{x}}_{G}\right).$$

When considering the cost‐benefit relationship of public goods for the group as a whole, which is equivalent to considering a group composed of a single player (N=1 and $p_{i}=\;1$), the relationship between collective investment and between‐group fitness is simply a quadratic function. This function captures the intuitive property of investment into public goods: more investment could potentially yield more benefits but, by sacrificing investment to the production of public goods, players necessarily reduce their ability to benefit from those goods. At thepeak of the quadratic, the optimum level of collective investment is:

(9) $$\theta =\frac{b-c}{2bc}.$$

Thus, the group has maximal between‐group fitness at some intermediate level of investment. In this way, a player's strategy is a compromise that reflects a trade‐off between fitness at different levels because within‐group fitness is maximized by no investment, whereas between‐group fitness is maximized by some intermediate level of investment.

The difference between what is good for the player and good for the group can be examined further in a simple analysis of “alignment” through the effects of parameter variation on the ESS (eq. 4) and coESS (eq. 3), where we consider the effect of varying each parameter when all other parameters are kept constant (Table 3). Although some parameters have the same effect on the quantity of investment by a player and quantity of collective investment by the group, such as the benefits (*b*) and the costs (*c*) of public goods, others have the opposite effects such as the number (*n*) and average frequency ($\bar{p}$) of contributors. This captures how players and groups experience a common trade‐off at the between‐group level over the benefits and costs of public goods, but there is a social dilemma about how players contribute toward public goods arising from the within‐group level (because of conflict between players strategies; see eq. 6). Consequently, increasing a contributor's frequency ($p_{i}$) increases their quantity of investment just as increasing the average frequency of contributors ($\bar{p}$) increases their quantity of collective investment, but increasing the average frequency of contributors ($\bar{p}$) decrease a contributor's quantity of investment. In this way, the conflict between players’ strategies can be considered as a conflict between levels (i.e., within‐ and between‐group fitness).

**Table 3:** The effect of increasing each parameter on the level of individual investment (${\hat{x}}_{i}$; see Eq. 4) and collective investment (${\hat{x}}_{G}$; see Eq. 3). The comparison is made keeping all other terms constant

Parameter	Individual investment ($x_{i}$)	Collective investment ($x_{G}$)
Benefit (*b*)	↑	↑
Cost (*c*)	↓	↓
Frequency ($p_{i}$)	↑	N/A†
Average contributor frequency ($\bar{p}$)	↓	↑
Number of contributors (*n*)	↑	↓

† This term does not directly appear within the expression for coESS of collective investment (Eq. 3).

## Discussion

To understand how players should strategically modulate their contributions toward group‐beneficial public goods in response to their social context, we analyzed how different genetic variants should contribute to public goods in relation to the number and frequencies of other genetic variants in the group. To simplify the discussion of our analyses, we describe the genetic variants to be the “players” in the game and consider their contributions to public goods as representing an “investment” they make in terms of the proportion of their potential fitness that they sacrifice to produce public goods (hence their potential fitness gives their total budget they can use to produce public goods). Most intuitively, and confirming the fundamental results of other public goods models (Frank 1996; Gavrilets 2015; Johnstone and Rodrigues 2016; Madgwick et al. 2018), a player should contribute a larger proportion of their fitness budget toward group‐beneficial behavior when they are at a higher frequency in a group. This is because a player at a higher frequency can realize more of the benefits from its own contribution toward public goods, making a larger investment more profitable. For a group composed of multiple players that contribute toward public goods, the level of collective investment made by all players in the group depends on the distribution of frequencies of the players within the group. Somewhat surprisingly, but following other models of facultative cooperation (Boyd and Richerson 1988; Pepper 2000; Gardner and Grafen 2009), we expect a group with more contributing players to contribute less toward group‐beneficial behavior. This outcome is a consequence of each contributor being at a lower frequency in the group, which disincentives each from investing (even in situations where they collectively have the same total frequency as a smaller number of contributors). Thus, within our model of strategic cooperation (i.e., not facultative cooperation because we consider quantitative contributions toward public goods), a group of players contribute more toward group‐beneficial behavior when the average frequency of all contributors is larger. Finally, and most surprisingly, we find that all contributors are expected to have equal transmission to the next generation irrespective of differences in their frequencies in the group. When contributors are at different frequencies in a group, this invariance pattern must reflect compensatory investment, where the contributors at higher frequency sacrifice a larger proportion of their potential fitness to produce public goods than those at lower frequencies, which wipes out any advantage from having a higher frequency. Invariance results have been demonstrated in evolutionary models of sex ratio and dispersal (e.g., Frank 1987; Rodrigues and Gardner 2016), but has not previously been identified in evolutionary models of public goods. Further, although analogous results to fitness invariance have been uncovered in previous economics models of public goods (Warr 1983; Bergstrom et al. 1986), we demonstrate that invariance can be evolutionarily stable and hence can be biologically relevant in the patterns of strategic cooperation.

Why have other similar evolutionary models of public goods (e.g., Frank 1996; Dionisio and Gordo 2006; Gavrilets 2015) not uncovered fitness invariance to frequency? The invariance result reflects the fact that our model considers fitness trade‐offs owing to allocation of limited resources to different fitness components (which is captured by our use of a multiplicative model of costs and benefits of public goods; see eq. 1). Fitness invariance across frequencies is a consequence of the presence of trade‐offs that favor players to quantitatively adjust their level investment into cooperation in a way that ends up cancelling out differences in their frequencies. This cancelling out arises from a function with trade‐offs in which cost and benefits have multiplicative effects on fitness, which will typically occur when they affect different fitness components (e.g., benefits come via an increase in fecundity, whereas costs come via a decrease in survival; see the antipredator vigilance scenario discussed in the Model and Results). Therefore, invariance is not necessarily expected in all possible biological scenarios (and hence would not necessarily emerge in all models of public goods). For example, models in which costs and benefits are a linear function of investment and have additive effects on fitness would not generate such fitness invariance. In this case, invariance cannot occur because the fitness function leads to facultatively all‐or‐none investment, with maximal investment (i.e., 100% of the resource budget) in public goods being favored whenever the benefits outweigh the costs and zero investment being favored when they do not. Likewise, although models where costs and benefits are nonlinear functions of investment but have additive effects on fitness can lead to quantitative levels of investment (not all‐or‐none) into cooperation, they would not produce fitness invariance (e.g., Gavrilets 2015). In contrast, other models of quantitative cooperation with multiplicative costs and benefits (e.g., Frank 1995) should generate fitness invariance, but this property has not been uncovered because they have not derived an informative analytical solution to players’ ESS that captures fitness invariance (though it has been speculated; e.g., Frank 1996). Our novel method of analysis based on the coESS has enabled fitness invariance to be uncovered, and indeed can be applied to other models to demonstrate fitness invariance (see Supporting Information 2 for an additive framing of our model using Hamilton's rule; see Supporting Information 3 where fitness invariance is found using the model from Frank 1995).

Fitness invariance to frequency is intriguing because it suggests that players are willing to pay more for public goods up to the level at which they come away with the same total payoff as all other contributors (in terms of transmission into the next generation), which means that those at lower frequencies sacrifice less of their fitness to produce public goods, while gaining the same benefits. This outcome reflects a property of investing into public goods that is analogous to the snowdrift game (Rapoport and Chammah 1966; Sugden 1986). In the classic snowdrift game, a player is favored to cooperate even when others do not, so an arbitrary asymmetry can cause players to adopt different roles (of contributor and free‐rider). In our model of strategic cooperation, the critical asymmetry is not arbitrary, but rather, reflects the fact that players are at different frequencies in a group. Those at a higher frequency have more to gain from each unit they contribute to public goods, and hence have more incentive to contribute. Logically, if players at higher frequencies contribute more than those at a lower frequencies, the frequency‐weighted fitness (which is their transmission into the next generation) asymmetry between players is diminished, which ultimately leads to an outcome where all contributors make contributions to public goods such that there is no remaining asymmetry. Interestingly, any free‐rider always has higher fitness than any contributor because they sacrifice none of their potential fitness to produce public goods, but gain the same benefits as the contributors. Therefore, in groups that contain contributors, players will have negative frequency‐dependent fitness, with free‐riders having the highest fitness and contributors having fitness in inverse order to their level of investment.

Given that the players are genetic variants that have information about their frequency in the group (such as a greenbeard or kin recognition gene), negative frequency‐dependent fitness could lead to the maintenance of polymorphism at the causal locus. Genetic recognition systems are most often discussed as providing a binary signaling system of match or mismatch with a single social partner, but we have introduced greater interaction complexity through quantitatively variable numbers/frequencies of players within a group and the potential to respond quantitatively through variable investment. As public goods give a fitness advantage to lower frequency players that free‐ride on the contributions of others (or otherwise invest less than other contributors), a new mutant that can differentiate itself as a player (presumably by producing a different signal) would potentially have a fitness advantage because of its low frequency. Consequently, even though all genetic variants are expected to play the same strategy (the ESS), negative frequency dependence arising from the quantitative adjustment of the level of cooperation within groups may lead to polymorphism by favoring rare genetic variants. In this way, strategic cooperation may explain how variation in social recognition persists, as a potential solution to Crozier's paradox (Crozier 1986; Rousset and Roze 2007). Further, although this is not something that we model (as we focus on the patterns of a player's ESS across social contexts), in longer term evolution, polymorphism may lead to worse outcomes for the production of public goods in groups, which may contain a large number of genetic variants at low frequencies, such that each may lack any incentive to be cooperative.

**Figure 1:**
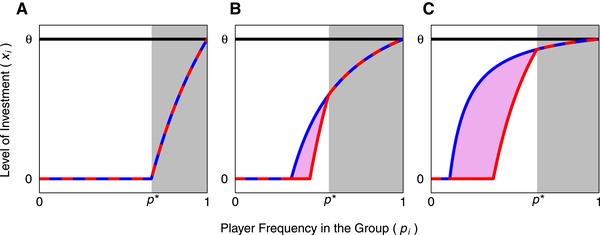
The ESS patterns of investment by a player into public goods across frequencies ($p_{i}$) under different cost‐benefit scenarios. In each panel, the red line indicates the ESS for the focal player in groups composed of two players (i.e., ${\hat{x}}_{i}$ when N=2) and the blue line the ESS for the focal player in groups that contain a large number of nonfocal players, each at low frequency. These lines overlap (indicated by the alternating red and blue dashed lines) when the focal players is at a frequency that is above the threshold p* (indicated by light‐gray shading), which is the frequency at which the focal player is the only contributor toward public goods. In regions where these two lines do not overlap, they set upper and lower limits to the expected level of investment (because players reduce investment in response to investment by others; groups with a large number of other players will set an upper limit on investment, whereas groups with two players set a lower limit on investment; see Eq. 6). The purple shaded area, therefore, indicates the range of possible patterns of investment by the focal player, with the exact value depending on the distribution of frequencies across nonfocal players within the group. The solid black horizontal line indicates the level of investment that maximizes group fitness (θ; Eq. 9). The three panels show patterns corresponding to different benefit‐to‐cost ratios (where costs were held constant at c=1 and benefits *b* were varied), which were chosen to capture three fundamental scenarios: (A) benefits relative to costs are low (b=3/2), such that players only contribute when they have a high frequency and consequently there is only ever a single contributor, (B) benefits relative to costs are high (b=3) and consequently there is potentially a small overlap between conditions where the focal player contributes and nonfocal players might also contribute, and (C) benefits relative to costs are very high (b=9) and consequently there is a very large region where nonfocal players may be motivated to contribute.

Another interesting implication of our analysis is that genetic variants may be favored to acquire information about other players’ frequencies. A player's best response to their group is not only determined by their own frequency but also by the investment by other players who are facing the same strategic dilemma. In different situations, a player may have the same frequency but benefit from showing different levels of investment; for example, a player benefits from higher investment into cooperation when there are two players as opposed to when there is a very large number of players (see Fig. 1). This hypothesis is interesting because greenbeard or kin recognition genes are typically discussed as self‐recognition systems, but our analysis suggests that recognition systems may be more generally favored if they can characterize the social context rather than only being able to provide information on their own presence or absence. However, it is challenging to identify a plausible biological mechanism that would allow players to detect different kinds of nonself. Therefore, the inability to recognize social context may constrain strategy evolution, such that our optimality analysis provides an important contrast to biological reality in systems where such recognition is not possible. It remains to be examined whether such strategic constraints might lead to better or worse outcomes for the production of public goods in groups.

The ESS pattern of cooperation through public goods is consistent with the expectation from kin selection theory, but our analysis reveals a role for conflict that is not present in the simplest statement of the conditions for cooperation that is captured by Hamilton's rule. Within this analysis, a player's frequency in the group is equated with their relatedness to the group. Although the level at which players contribute to public goods reflects the relatedness‐dependent profitability of such contributions (in terms of the cost‐benefit analysis expressed in Hamilton's rule), players are expected to reduce their own contribution below the simple cost‐benefit analysis when other members of their group are favored to contribute toward public goods. This outcome captures the role of conflict in cooperation through group‐beneficial public goods, where players potentially contribute at a level that is lower than expected under the strict profitability analysis of Hamilton's rule. These same results can be understood from a levels of selection perspective, where contributions to public goods can reduce a player's within‐group fitness because they sacrifice a component of their potential fitness by contributing toward public goods, which can leave them with lower relative fitness compared to members of their group who contribute less. As a result, players maximize their within‐group fitness by contributing less than others, which can drive down the incentive to contribute. However, contributions to public goods benefit players through their impact on the fitness of the group (because all members of a group gain the same benefits) and therefore the ESS level of investment in public goods will reflect the disincentivizing effect of within‐group fitness and the incentivizing effect of between‐group fitness.

Associate Editor: A. Charmantier

## Supporting information

Supporting Information
