# Supplementary material for: Evolution of strategic cooperation
Source: Evol Lett. 2020 Feb 25;4(2):164–75. doi: 10.1002/evl3.164 (PMC7156107; doi:10.1002/evl3.164)
Supplement: Supplementary file 1 — Supporting Information [file EVL3-4-164-s001.docx]

Supplement for

*Evolution of Strategic Cooperation*

Philip G. Madgwick and Jason B. Wolf

Email: pgmadgwick@gmail.com

**Sections in the supplement:**

1. Solving the evolutionarily stable strategy
2. Deriving a marginal Hamilton’s rule
3. An invariance result in a similar model

## **Solving the evolutionarily stable strategy**

In the main-text, the evolutionarily stable strategy (ESS) for a player was derived by utilising the coevolutionarily stable strategy (coESS) for the group, which we explain in further detail here. We develop the novel concept of the coESS (but see *e.g.* Eshel 1985 for a similar previous usage of this term) to provide a means of deriving optimal patterns of cooperation through public goods for a group of players. The use of the coESS is necessary because of the difficulty of analysing a $N$ player public goods game, where $N$ could refer to any number of players that could also have any distribution of frequencies. The quantity of investment by each player in any possible type of group can be analytically solved, but it such a solution would require a very large number of conditional expressions (and would not be generalisable to other scenarios). However, by using the coESS, we can derive an informative analytical description of the ESS. The coESS describes the total investment into public goods across the group, which we can derive based upon group-level variables that have known properties that can inform their analysis.

We provide a detailed solution to the ESS, using a specific notation with respect to a focal player ($i$) and a combined set of non-focal players(s) ($-i$). Using this notation, the collective investment by the group would be written as the sum of both the focal player’s investment and any investment arising from others: $x_{G}=p_{i}x_{i}+p_{-i}x_{-i}$. This notation is useful because it allows us to ignore the details of the number and frequencies of other players. The ESS is solved by starting from the optimal strategy that maximizes fitness – *i.e.*, a player’s ‘best-response’ ($\vec{x}_{i}$), which is dependent upon the level of investment made by other players. Players are assumed to have access to perfect information about their frequency in the group ($p_{i}$) and the investment from all other players ($p_{-i}x_{-i}$) and so, given that strategies are restricted to within the range $0\leq\vec{x}_{i}\leq1$, the best-response is solved by setting the derivative $d\omega_{i}/d\vec{x}_{i}=0$ (where the collective investment can be written as $x_{G}=p_{i}\hat{x}_{i}+p_{-i}\hat{x}_{-i}$):

| $0=bp_{i}^{2}-cp_{i}-bcp_{i}p_{-i}x_{-i}-2bcp_{i}^{2}\vec{x}_{i}$  $\vec{x}_{i}=\frac{1}{2}\left[ \frac{1}{c}-\frac{1}{p_{i}b}-\frac{p_{-i}x_{-i}}{p_{i}} \right]$ | (S1.1) |
| --- | --- |

The best-response is the same as the evolutionarily stable strategy (ESS; $\hat{x}_{i}$) when all other players are also playing their best-response (*i.e.* other players are also playing the ESS: $\hat{x}_{-i}$).

The ESS can be resolved by from the best-response equation by substituting in the best-response of the other players, which can be easily resolved in a two-player game by solving a second player’s ($-i$) best response using $p_{-i}=1-p_{i}$:

| $\vec{x}_{-i}=\frac{1}{2}\left[ \frac{1}{c}-\frac{1}{(1-p_{i})b}-\frac{p_{i}x_{i}}{1-p_{i}} \right]$ | (S1.2) |
| --- | --- |

Thus, the path to solving the ESS ($\hat{x}_{i}$) for two-players involves substituting Equation (S1.2) into Equation (S1.1):

| $\vec{x}_{i}=\frac{1}{2}\left[ \frac{1}{c}-\frac{1}{p_{i}b}-\frac{1}{2p_{i}}\left[ \frac{1-p_{i}}{c}-\frac{1}{b}-p_{i}\vec{x}_{i} \right] \right]$  $4p_{i}\vec{x}_{i}=\frac{2p_{i}}{c}-\frac{2}{b}-\frac{1-p_{i}}{c}+\frac{1}{b}+p_{i}\vec{x}_{i}$  $\vec{x}_{i}=\frac{\left( 3p-1 \right)b-c}{3p_{i}bc}$ | (S1.3) |
| --- | --- |

However, this solution does not represent the ESS because the value given by Equation (S1.2) allows for negative investment (as if $\vec{x}_{-i}<0$), which is not possible. Consequently, Equation (S1.3) is a solution to a part of the ESS when $\vec{x}_{-i}>0$. When $\vec{x}_{-i}<0$, it follows that $p_{-i}\vec{x}_{-i}=0$, so the second part of the ESS for the first player is:

| $\vec{x}_{i}=\frac{1}{2}\left[ \frac{1}{c}-\frac{1}{p_{i}b} \right]$  $\hat{x}_{i}=\frac{p_{i}b-c}{2p_{i}bc}$ | (S1.4) |
| --- | --- |

Thus, overall, the ESS must be written as a set of conditional expressions. Note that either expression could present the case where $\hat{x}_{-i}=0$ but for clarity we have opted for mutually exclusive conditions:

| $\hat{x}_{i}=\begin{matrix} \vec{x}_{-i}\geq0 & \begin{matrix} \vec{x}_{i}\geq0 & \frac{\left( 3p-1 \right)b-c}{3p_{i}bc} \\ \vec{x}_{i}<0 & 0 \end{matrix} \\ \vec{x}_{-i}<0 & \begin{matrix} \vec{x}_{i}\geq0 & \frac{p_{i}b-c}{2p_{i}bc} \\ \vec{x}_{i}<0 & 0 \end{matrix} \end{matrix}$ | (S1.5) |
| --- | --- |

This same technique of resolving the ESS using conditional expressions could be used to solve situations with more than two players, but the number of potential combinations of players investing ($\hat{x}_{i}\geq0$) or not ($\hat{x}_{i}<0$) increases exponentially with the number of players (i.e., at a rate of $2^{N}$^)^, meaning that the ESS is described by an ever larger set of conditional expressions – which precludes any informative analysis. Consequently, although this method can exactly solve the ESS in groups with a particular number of players, it cannot provide an informative expression for groups of any number of players.

To solve the ESS, the expression in equation (S1.1) must be resolved simultaneously for all players, which we define as collectively behaving as per the coevolutionarily stable strategy (coESS; $\hat{x}_{G}$) for the group: the coESS refers to a collective level of investment into public goods by the group of players that perform the ESS, which leads each player to contribute a level of investment that depends on their frequency ($p_{i}$). When the number of players is not specified, we do not believe that it is possible to solve the investment by a given player across all frequency combinations, which is supported by the logic that each number of players and frequency distribution has its own set of conditional expressions as its solution. Instead we use a novel method to discern the critical variables underlying how a player’s strategy changes in response to their own frequency and the number and frequency distribution of other players.

At the equilibrium where all players display the conditional ESS, not all players necessarily contribute toward the coESS because some players have too low a frequency to benefit from investment into public goods. Players in any given social context can be divided into two groups at the coESS: a set of $n$ ‘contributors’ that contribute toward collective investment and a set of $N-n$ free-riders that do not contribute (where $N$ is the number of players in the group). Formally, given that the best-response (eqn. S1.1) can be negative (despite the fact that players cannot actually display negative investment), the set of $n$ contributors includes all players that have a non-negative investment in the ESS ($\hat{x}_{i}\geq0$), which includes the non-investing strategy where $\hat{x}_{i}=0$ (which turns out to be important because players that have this strategy are included in the invariance result). Therefore, players can be divided into two classes based on Equation S1.1: ‘contributors’ that have investment values $\hat{x}_{i}\geq0$ and free-riders that have an optimal investment below zero $\hat{x}_{i}<0$, but which is constrained to be zero. However, for simplicity and given that the frequency where $\hat{x}_{i}=0$ exactly is infinitesimally small, we can more generally describe players as one of $n$ contributors ($\hat{x}_{i}>0$) or $N-n$ free-riders (whose real strategy is constrained to $\hat{x}_{i}=0$) without worrying about this distinction (as we do in the main text) – and we use the terms contributors and free-riders going forward.

By taking the average of the frequency of all contributors $\bar{p}=\frac{1}{n}\sum_{j=1}^{n} p_{j}$, the collective frequencies of contributors is $n\bar{p}$, whilst the collective frequencies of free-riders is $1-n\bar{p}$. Thus, the coESS can be calculated as the sum of the contributions by ESS contributors:

| $\hat{x}_{G}=\sum_{i=1}^{n} p_{i}\hat{x}_{i}$  $=\sum_{i=1}^{n} \frac{1}{2}\left[ \frac{p_{i}}{c}-\frac{1}{b}-p_{-i}\hat{x}_{-i} \right]$  $=\frac{1}{2}\left[ \frac{n\bar{p}}{c}-\frac{n}{b} \right]-\sum_{i=1}^{n} \frac{p_{-i}\hat{x}_{-i}}{2}$ | (S1.6) |
| --- | --- |

Given that $\hat{x}_{G}=p_{i}\hat{x}_{i}+p_{-i}\hat{x}_{-i}$ by definition, the act of summing $p_{-i}\hat{x}_{-i}$ with respect to the $i$^th^ focal player $n$ times is the same as $\sum_{i=1}^{n} {\hat{x}_{G}-p}_{i}\hat{x}_{i}=n\hat{x}_{G}-\sum_{i=1}^{n} p_{i}\hat{x}_{i}=n\hat{x}_{G}-\hat{x}_{G}$, so this leads to:

| $\hat{x}_{G}=\frac{1}{2}\left[ \frac{n\bar{p}}{c}-\frac{n}{b}-\hat{x}_{G}(n-1) \right]$  $=\left[ \frac{1}{n+1} \right]\left[ \frac{n\bar{p}}{c}-\frac{n}{b} \right]$  $=\frac{n(\bar{p}b-c)}{\left( n+1 \right)bc}$ | (S1.7) |
| --- | --- |

Equation (S1.7) corresponds to Equation (3) in the main-text. As the coESS reflects ESS behaviour for a group of investing players, we can substitute the coESS into the best-response (eqn. S1.1), using $p_{-i}\hat{x}_{-i}=\hat{x}_{G}-p_{i}\hat{x}_{i}$, to resolve the ESS:

| $\hat{x}_{i}=\frac{1}{2}\left[ \frac{1}{c}-\frac{1}{p_{i}b}-\frac{\hat{x}_{G}-p_{i}\hat{x}_{i}}{p_{i}} \right]$  $=\frac{1}{c}-\frac{1}{p_{i}b}-\frac{\hat{x}_{G}}{p_{i}}$  $=\frac{1}{c}-\frac{1}{p_{i}b}-\left[ \frac{n(\bar{p}b-c)}{\left( n+1 \right)p_{i}bc} \right]$  $=\frac{p_{i}b-c-n(\bar{p}-p_{i})b}{p_{i}\left( n+1 \right)bc}$ | (S1.8) |
| --- | --- |

Equation (S1.8) corresponds to Equation (4) in the main-text. Given that players’ investments are constrained to be non-negative ($\hat{x}_{i}\geq0$), these solutions only represent the quantity of investment at the ESS if players can be classified as being one of the $n$ contributors or $N-n$ free-riders. A player’s strategy can be categorised based on their frequency by solving the boundary condition where individuals would switch from being a free-rider to a contributor, which corresponds to the value where $\hat{x}_{i}=0$ (using eqn. S1.8). Given that a player is a contributor when $\hat{x}_{i}=0$, a player can be classified as an contributor based on their frequency when:

| $p_{i}\leq\frac{\bar{p}nb+c}{b(n+1)}$ | (S1.9) |
| --- | --- |

Equation (S1.9) corresponds to an equation found in Table 2 of the main-text. Because this limit is necessarily self-referential to the average frequency and number of contributors ($\bar{p}, n$), the ESS (eqn. S1.8) represents a closed-solution only insofar as players have been classified as contributors or free-riders. This problem can be solved numerically for any situation without needing to solve the conditional expressions that exactly describe an ESS. For this we propose an algorithm for classifying players where they are ranked in terms of their frequency (high to low) and then each player is ‘tested’ in turn to examine whether or not they have a non-negative investment strategy in the new social context (*i.e.* when that player is included in the calculation of the average frequency and number of contributors; $\bar{p}, n$). If the player has a non-negative investment strategy then they are a contributor and the next highest frequency player can then be tested. The algorithm can stop searching for new players to add into the contributor class once the next highest frequency player fails to have a positive investment. At this point, the candidate player is not added into the contributor class and the ESS is fully resolved for this particular scenario with the previous calculations of the average frequencies and number of contributors prior to the current test. This method can be much more time efficient than solving the conditional expressions that exactly describe an ESS (testing at most $N$ equations rather than deriving $2^{N}$ expressions).

## **Deriving a marginal Hamilton’s rule**

Although we discuss numerous similarities between inequalities utilising the ESS and Hamilton’s rule in the main text, their formal relationship is complicated. Under quantitative strategies, we can use Hamilton’s rule to solve the ESS, but the rule must be framed in a marginal form (Taylor and Frank 1996; Frank 2013) in which, given relatedness ($r$), describes the ESS as the equalisation of the specially-constructed marginal costs ($C_{m}$) and marginal benefits ($B_{m}$) of cooperation: $rB_{m}-C_{m}=0$. We say ‘specially-constructed’ to emphasise that these marginal costs and benefits ($C_{m}, B_{m}$) are not the same as the cost and benefit terms ($c, b$) in our model, and so we have to calculate them in the terms of our existing model. The two differ because the cost and benefit terms in our model are not defined with respect to fitness, but rather, are parameters that interact (multiplicatively) to determine fitness, whereas the cost and benefit terms that are relevant to Hamilton’s rule are defined in terms of additive components of fitness. In this way, the marginal version of Hamilton’s rule we present here captures the relationship between the models that assume additivity of costs and benefits and our model in which they are multiplicative. Because there are trade-offs between different fitness components caused by the allocation of limited resources, the costs and benefits of contributing to public goods are necessarily multiplicative in our model, but in any given scenario we can derive an additive expression (the marginal Hamilton’s rule) that expresses the costs and benefits in an additive form. In doing so, we are able to use our model to address questions that are typically posed in additive models, such as ‘should individuals contribute to public goods given the current social context – *i.e.* given *r* and the associated costs and benefits?’.

In-keeping with our model, we can rearrange the ESS (eqn. 5), which has been solved by taking the derivative of fitness with respect to the level of investment in public goods ($d\omega_{i}/dx_{i}$), given an equilibrium quantity of collective investment by the group ($\hat{x}_{G}$), into the marginal form:

| $p_{i}b-c-bc(p_{i}\hat{x}_{i}+\hat{x}_{G})=0$ | (S2.1) |
| --- | --- |

On the right-hand-side, the first two parts, $p_{i}b-c$, are equivalent to the classic form of Hamilton’s rule (Charnov 1977), where a non-zero investment strategy is favoured if the product of the benefit from investment ($b$) and frequency/relatedness ($p_{i}$) outweighs the private cost ($c$). It is important to keep in mind that $p_{i}$ does not represent the relatedness of a player to other members of a group, but instead represents ‘whole-group relatedness’ (Hamilton 1975; Pepper 2000), so it includes the relatedness of a player to itself (since it is a member of the group). The third part of this marginal Hamilton’s rule, $-bc(p_{i}\hat{x}_{i}+\hat{x}_{G})$, is equivalent to other derivations of Hamilton’s rules that consider the synergistic impact of the social context on fitness (Queller 1994; Frank 1998), which, in our model, reflects how a focal player’s motivation to invest into public goods declines both in response to increasing their own level of investment ($p_{i}\hat{x}_{i}$) and the investment afforded all players within the group including themselves ($\hat{x}_{G}$). From a single player’s perspective, this means that increasing their own investment into public goods by some marginal unit of resources has a two-fold effect on disincentivising any further investment by that player, which stems from that player shifting the unit of resource both toward the production of public goods (and hence incentivising them to exploit public goods more) and away from the utilization of public goods (and hence decreasing their receipt of the benefits from public goods and thereby incentivising them to exploit public goods more again).

In the marginal Hamilton’s rule, the social context of investment provided by others is a property of both the marginal costs ($C_{m}$) and marginal benefits ($B_{m}$) of cooperation. Thus, ignoring the connection between personal ($\hat{x}_{i}$) and collective ($\hat{x}_{G}$) investment (see Supplement 1), the marginal form of Hamilton’s rule can be written by grouping the relatedness-weighted (*i.e.* multiplied by $p_{i}$) benefits and the relatedness-independent costs:

| $p_{i}b(1-\hat{x}_{i})=c(1+b\hat{x}_{G})$ | (S2.2) |
| --- | --- |

which implies that $B_{m}=b(1-\hat{x}_{i})$ and $C_{m}=c(1+b\hat{x}_{G})$. By including additional factors beyond the direct costs and benefits of strategic behaviour, the marginal Hamilton’s rule can be used to derive a solution to the ESS (Grafen 1985; Queller 1992; Taylor and Frank 1996; Frank 2013). However, because of the connection between personal ($\hat{x}_{i}$) and collective ($\hat{x}_{G}$) investment, this method only solves the conditional expressions of the ESS and cannot by itself be used to derive the informative analytical solution with utilising the coESS (Supplement 1).

## **An invariance result in a similar model**

Fitness invariance to frequency has not been uncovered in other evolutionary models of public goods, but is likely to be a robust finding in models that consider trade-offs caused by the allocation of limited resources to public goods (and hence, which assume that costs and benefits are multiplicative). To demonstrate this, we apply our modelling approach to examine the model from Frank (1995), which forms the basis for several other models (*e.g.* Frank 1996, 2010; Gardner and West 2004; Dionisio and Gordo 2006, 2007; El Mouden et al. 2010). The difference between our model and Frank’s (1995) model is the nature of public goods – whether they are a production or appropriation (see Cornes and Sandler 1986). Our model uses a ‘production’ framework, where individuals invest into the creation of new public goods. Frank’s (1995) model uses an alternative ‘appropriation’ framework, where individuals compete for a fixed quantity of pre-existing public goods, and hence they invest into their ability to compete for those goods. Here we show that, despite the conceptual and mathematical differences between the models, the results from Frank (1995) are broadly consistent in terms of their predictions for the evolution of strategic cooperation, including the invariance result. However, although the basic findings from appropriation-style models are similar to what we find for our production-style model, we also emphasise the utility in adopting our model for the analysis of strategic cooperation due to features that make the appropriation-style model harder to analyse.

Frank’s (1995) model imagines that some total resource is being divided up between the players in the group based on their competitive ability, but that the quantity of those resources is reduced by the competitiveness of the group. This creates a trade-off between a cost and benefit function that is analogous to that seen in our production-style model. Frank’s (1995) model can be described using two parameters: the average competitive intensity of a group ($z_{i}$) and the competitive intensity the $j$-th individual in the $i$-th group ($z_{ij}$). Based on these parameters, fitness is given by:

| $\omega_{ij}=(z_{ij}/z_{i})(1-z_{i})$ | (S3.1) |
| --- | --- |

Frank (1995) solves the model using optimality assumptions, in maximizing $\omega_{ij}$ with respect to $z_{ij}$ given some level of relatedness ($r$) among individuals in the group. The evolutionarily stable strategy of competition at equilibrium is $z_{ij}^{*}=1-r$. Hence, when a player is the only member of a group ($r=1$; $N=1$) the evolutionarily stable strategy (ESS) is for a player to allocate zero investment toward competition because there are no competitors to compete against. In contrast, in our production-style model, the same situation would favour a player who produced public goods at the group-optimal level ($\varphi$; see eqn. 3). Thus, the trade-offs are slightly different in the two styles of public goods model: our production-style model includes an explicit trade-off in the costs and benefits of public goods production (hence an intermediate optimum), whereas the Frank’s (1995) production-style model considers the trade-off between cooperation and competition over the utilization of a fixed resource.

Although our production-style model and Frank’s (1995) appropriation-style model are built on different frameworks, their conceptual differences can be shown to be trivial by making two simple alterations to each expression of the public goods game to demonstrate their similarity. First, we can ‘invert’ our model to both switch from production- to appropriation-style public goods and to remove the trade-off between the costs and benefits of producing public goods. This can simply be done by replacing the terms like for like, such that the costs of public goods ($-c$) change to the individual-advantage of competition ($+c$), whilst the benefits of public goods ($+b$) changes to the group-disadvantage of collective competition ($-b$). All other features of the derived ESS, coESS and other such results as described in the main-text are equivalent given this substitution. However, it is important to note that, in switching from strategies of investment ($x_{i}$) to strategies of competition ($x_{i}'$), the scale of $x_{i}'$ is reversed compared to $x_{i}$(*i.e.* $x_{i}'=1-x_{i}$) resulting in an inverted shape for the ESS, so a player that is a ‘full competitor’ ($x_{i}'=1$) is akin to a non-investing ‘full cheater’ ($x_{i}=0$) in the alternative game form.

Second, we can introduce frequency (here given as $r_{i}$) into Frank’s (1995) model in the same way as in our model, rather than examining the relatedness between individuals. This amounts to taking a direct-fitness approach rather than (as Frank 1995 does) considering a player’s inclusive fitness (*i.e.* both direct and indirect fitness). Here, we use the notation $z_{i}$ to represent the competitive strategy of the $i$-th player, $1$ is the resource to be divided and $z_{G}$ is the cost of collective competition among group members. Thus, in Frank’s (1995) model the expression for fitness can incorporate resources by weighting each player’s effects on collective competition ($z_{G}=\sum_{j=1}^{N} r_{j}z_{j}$) to yield:

| $\omega_{i}=(r_{i}z_{i}/z_{G})(1-z_{G})$ | (S3.2) |
| --- | --- |

Given some level of investment by other (*i.e.* not $i$-th) player(s), which we denote $r_{-i}z_{-i}$ (see Supplement 1), the best-response is then:

| $\vec{z}_{i}=\left( 1/r_{i} \right)\left( -r_{-i}z_{-i}+\sqrt{r_{-i}z_{-i}} \right)$ | (S3.3) |
| --- | --- |

There is no generalised solution to the coESS because the second term of the best-response does not simplify, so the simplest expression is: $\hat{z}_{G}=\frac{1}{n}\left( 1-n\bar{r}+\sum_{j=1}^{n} \sqrt{\hat{z}_{G}-r_{j}\hat{z}_{j}} \right)$ where $n\bar{r}$ has parity to $n\bar{p}$ in our model. However, in the special case where each player is not a full competitor (so $\hat{z}_{i}<1$; ergo $N=n$), the coESS is:

| $\hat{z}_{G}=1-\frac{1}{n}$ | (S3.4) |
| --- | --- |

Within this special case, the ESS is given as:

| $\hat{z}_{i}=\frac{\hat{z}_{G}(1-\hat{z}_{G})}{r_{i}}$  $=\left( \frac{1}{r_{i}} \right)\left( \frac{1}{n}-\frac{1}{n^{2}} \right)$ | (S3.5) |
| --- | --- |

Comparing the coESSs (eqn. 6 in main-text and eqn. S3.4 here) and ESSs (eqn. 7 in main-text and eqn. S3.5 here) between models reveals that both have a similar relationship with respect to the number of players contributing to the coESS ($n$) and the frequency of a focal player ($r_{i}$). Yet, even in the inverted form of our model there are additional terms that relate to the advantages and disadvantages of competition, which are explicitly absent in Frank’s (1995) model, which includes no such constants (but see Frank 2010 for a generalised framework that could include these constants).

Interestingly, although it has not been explicitly examined in Frank’s (1995) or related models, both these models show the fitness invariance result that we find for the our production-style model, where investing/competing players vary their strategy in such a way that they obtain equal fitness (which Frank 1996 suspected but did not prove). This can be observed by substituting the ESS (eqn. S3.5) into the expression for an individual player’s fitness (eqn. S2.2), which yields $\omega_{i}={(1-z_{G})}^{2}$. The fitness expression does not include the focal player’s frequency ($r_{i}$), hence their fitness is invariant to their frequency and, consequently, investing/competing players obtain equal fitness. In demonstrating the consistency between the main result of our model and equivalent forms of Frank’s (1995) model, we have shown that both forms of the public goods game have similar properties, which emphasises the generality of conclusions made in either game (with respect to the other). Consequently, Frank’s (1995) model also describes the same tension between within- and between-group competition underlying the outcomes for group fitness (Frank 2010), has an equivalent role for frequency or relatedness (Frank 1995, 2003, 2009) and shows that there is higher investment into public goods from better endowed individuals than individuals with fewer resources (Frank 1996). Yet, due to Frank’s (1995) intended analysis having a different focus, there are factors that are simpler to examine through Frank’s (1995) model (*e.g.* fixed private benefits, or alternative synergistic relationships between individual behaviour and so on) but equally Frank’s (1995) model lacks an informative expression for the ESS (*i.e.* the coESS in eqn. S3.3 does not resolve to something that can be analysed) and is therefore more difficult to use to analyse the logic of strategic cooperation – or indeed to demonstrate invariance.

## **References**

Charnov, E. L. 1977. An elementary treatment of the genetical theory of kin-selection. Journal of Theoretical Biology 66:541–550.

Cornes, R., and T. Sandler. 1986. The Theory of Externalities, Public Goods, and Club Goods. Cambridge University Press.

Dionisio, F., and I. Gordo. 2006. The tragedy of the commons, the public good dilemma and the meaning of rivalry and excludability in evolutionary biology. Evolutionary Ecology Research 8:321–332.

———. 2007. Controlling excludability in the evolution of cooperation. Evolutionary Ecology Research 9:365–373.

El Mouden, C., S. A. West, and A. Gardner. 2010. The enforcement of cooperation by policing. Evolution 64:2139–2152.

Eshel, I. 1985. Evolutionary Genetic Stability of Mendelian Segregation and the Role of Free Recombination in the Chromosomal System. The American Naturalist 125:412–420.

Frank, S. A. 1995. Mutual policing and repression of competition in the evolution of cooperative groups. Nature 377:520–522.

———. 1996. Policing and group cohesion when resources vary. Animal Behaviour 52:1163–1169.

———. 1998. Foundations of Social Evolution. Princeton University Press.

———. 2003. Repression of competition and the evolution of cooperation. Evolution 57:693–705.

———. 2009. Evolutionary Foundations of Cooperation and Group Cohesion. Pages 3–40 *in* S. Levin, ed. Games, Groups, and the Global Good. Springer Physica-Verlag Berlin Heidelberg 2009.

———. 2010. A general model of the public goods dilemma. Journal of Evolutionary Biology 23:1245–1250.

———. 2013. Natural selection. VII. History and interpretation of kin selection theory. Journal of Evolutionary Biology 26:1151–1184.

Gardner, A., and S. A. West. 2004. Cooperation and Punishment, Especially in Humans. The American Naturalist 164:753–764.

Grafen, A. 1985. A geometric view of relatedness. Oxford Surveys in Evolutionary Biology 2:28–89.

Hamilton, W. D. 1975. Innate Social Aptitudes of Man: an approach from evolutionary genetics. Pages 133–153 *in* R. Fox, ed. ASA Studies 4: Biosocial Anthropology. Malaby Press.

Pepper, J. W. 2000. Relatedness in trait group models of social evolution. Journal of Theoretical Biology 206:355–368.

Queller, D. C. 1992. Quantitative Genetics, Inclusive Fitness, and Group Selection. The American Naturalist 139:540–558.

———. 1994. Genetic relatedness in viscous populations. Evolutionary Ecology 8:70–73.

Taylor, P. D., and S. A. Frank. 1996. How to make a kin selection model. Journal of Theoretical Biology 180:27–37.
